# Oral Zinc Supplementation in Acne Vulgaris and Hidradenitis Suppurativa: An Updated Comprehensive Review

**DOI:** 10.3390/nu18172865

**Published:** 2026-09-02

**Authors:** Sabrina Spiniello, Davide Termini, Gianluca Tavoletti, Gianluca Avallone, Silvia Alberti-Violetti, Angelo Valerio Marzano, Gianluca Nazzaro

**Affiliations:** 1 Dermatology Unit, Fondazione IRCCS Ca’ Granda Ospedale Maggiore Policlinico, 20122 Milan, Italyangelo.marzano@unimi.it (A.V.M.);; 2 Department of Pathophysiology and Transplantation, Università Degli Studi di Milano, 20122 Milan, Italy

**Keywords:** acne vulgaris, hidradenitis suppurativa, micronutrients, antioxidants, skin diseases, zinc, antibiotic-sparing strategies

## Abstract

Oral zinc supplementation has emerged as a potential ⁠adjunctive strategy in inflammatory follicular disorders, including ⁠acne vulgaris and hidradenitis suppurativa. Beyond its role as an essential micronutrient, zinc modulates several pathways and biological processes directly ⁠relevant to these diseases, including innate immunity, oxidative stress, neutrophil-driven inflammation, antimicrobial defense, wound repair, and possibly sebaceous gland activity and androgen metabolism. In acne, available data support a potential clinical benefit, mainly on inflammatory lesions, with ⁠potential relevance in antibiotic-sparing approaches. In hidradenitis suppurativa, the evidence remains more limited and is largely based on small observational or uncontrolled studies, although a possible adjunctive role has been suggested in mild-to-moderate disease and maintenance therapy. However, differences across studies in zinc formulation, ⁠dose, treatment duration, baseline zinc assessment, and outcome measures make it difficult to draw firm conclusions, and further randomized controlled trials are needed to identify which patients are most likely to benefit. Considering the available evidence, zinc represents an accessible and clinically attractive adjunct in acne vulgaris and hidradenitis suppurativa. This review critically appraises the evidence ⁠on oral zinc in acne vulgaris and hidradenitis suppurativa, with particular attention to biological plausibility, clinical effectiveness, dosing and safety.

## 1. Background

Acne vulgaris and hidradenitis suppurativa (HS) are common chronic inflammatory skin diseases that originate from distinct components of the follicular apparatus. Acne primarily affects the pilosebaceous unit, whereas HS is considered a disorder of the follicular epithelium [1,2,3,4,5].

Acne vulgaris is a chronic inflammatory disorder of the pilosebaceous unit characterized by follicular hyperkeratinization, increased sebum production, *Cutibacterium acnes* (*C. acnes*) proliferation, and a dysregulated innate and adaptive immune response. Clinically, it presents with a spectrum of lesions ranging from comedones and inflammatory papules to pustules, nodules, and scars, predominantly affecting the face, chest, and back. Current treatment options include topical retinoids, benzoyl peroxide, antibiotics, hormonal therapies, and oral isotretinoin. While isotretinoin remains the most effective treatment for severe acne and can induce prolonged remission, its use requires careful clinical and laboratory monitoring and, in women of childbearing potential, strict adherence to pregnancy prevention measures because of its well-established teratogenicity. Furthermore, tolerability issues, contraindications, and the growing concern of antimicrobial resistance continue to limit the long-term use of several conventional therapies [6].

HS, also referred to as “acne inversa”, is a chronic inflammatory disease of the hair follicle associated with follicular occlusion and dysregulated immune pathways. Current therapeutic strategies encompass antibiotics, retinoids, biologic agents, and surgery, but outcomes are often unsatisfactory and limited by adverse effects and poor tolerability [7].

Dietary factors and micronutrient imbalances have been increasingly investigated as potential modulators of acne and HS [8,9,10,11]. In particular, reduced serum zinc levels have been reported in both conditions, raising interest in the potential role of zinc status in disease activity and zinc supplementation as an adjunct to conventional treatment [12,13]. These observations have stimulated growing interest in targeted nutritional interventions, including zinc supplementation, as adjuncts to conventional medical treatment.

Zinc is an essential trace element involved in numerous biological processes, including immune regulation, antioxidant defense, and tissue repair [14,15]. These properties have supported longstanding interest in its use in inflammatory skin diseases [16].

Interest in zinc as a therapeutic option for acne dates back to the 1970s, when preliminary observations suggested a potential benefit of oral supplementation [17]. More recent clinical studies and evidence syntheses have renewed interest not only in acne but also in HS.

This narrative review provides an updated overview of oral zinc supplementation in the management of acne vulgaris and HS. It explores the biological rationale underlying zinc’s therapeutic effects and offers a critical appraisal of the available evidence, including both landmark studies and recent data, with particular emphasis on the strength and consistency of the findings.

## 2. Methods

This manuscript was designed as a narrative review aimed at providing an updated and critical overview of the available evidence on oral zinc supplementation in acne vulgaris and hidradenitis suppurativa (HS). A literature search was conducted to identify relevant clinical, translational, and experimental studies investigating the role of zinc in these inflammatory follicular disorders. Given the narrative nature of the review, the literature search was not conducted according to a predefined protocol or formal systematic review reporting framework. Particular attention was given to peer-reviewed studies providing clinical outcomes, mechanistic insights, safety data, and information on zinc dosage and treatment duration.

## 3. Biological Functions of Zinc in Skin Homeostasis and Immune Regulation

Zinc is a cofactor for over 300 enzymes and more than 2000 transcription factors, highlighting its fundamental role in cellular proliferation, differentiation, and immune regulation [15]. Within the skin, zinc participates in several biological processes relevant to the pathogenesis of both acne and HS. Evidence supporting these effects derives from a combination of experimental studies, clinical investigations in acne vulgaris and HS, and broader research on zinc biology, inflammation, and wound healing. One of the best-characterized functions of zinc is its antioxidant activity. Through enzymes such as copper/zinc superoxide dismutase, zinc contributes to the detoxification of superoxide radicals and limits the accumulation of reactive oxygen species [18]. In parallel, zinc promotes the synthesis of metallothioneins, cysteine-rich proteins with potent free radical scavenging activity and reduces oxidative damage by competing with redox-active metals such as iron and copper at critical cellular sites [19]. In addition to its antioxidant effects, zinc exerts broad immunomodulatory and anti-inflammatory effects. Clinical and experimental studies have demonstrated reductions in systemic inflammatory markers, including C-reactive protein (CRP) and tumor necrosis factor alpha (TNF-α) [20]. In addition, experimental studies suggest that zinc modulates innate immune responses through regulation of Toll-like receptor 2 (TLR-2), a key mediator of microbial recognition and inflammatory signaling expressed by keratinocytes and immune cells. By attenuating TLR-2 expression and downstream signaling pathways, zinc may reduce the production of pro-inflammatory mediators and contribute to the suppression of cutaneous inflammation [21]. Experimental data further indicate that zinc can inhibit neutrophil chemotaxis and reduce cytokine release, mechanisms that may help limit the excessive inflammatory response involved in tissue destruction and sinus tract formation [22].

Antimicrobial activity may further contribute to its therapeutic potential. Experimental and clinical studies conducted in patients with acne have shown inhibitory effects against *C. acnes* and other skin-associated microorganisms, while also suggesting synergistic interactions with selected antibiotics that could enhance antimicrobial efficacy and support antibiotic-sparing strategies [23,24]. In addition, zinc has been reported to inhibit bacterial biofilm formation in vitro, a property that may have potential relevance in inflammatory skin disorders [25].

Additional mechanisms may involve modulation of androgen metabolism. In vitro studies have demonstrated inhibition of 5α-reductase, the enzyme responsible for the conversion of testosterone into dihydrotestosterone, thereby providing a potential indirect anti-androgenic effect. By influencing sebaceous gland activity and sebum production, this mechanism may contribute to the clinical benefits observed in acne and, potentially, in HS, where androgens are also thought to be involved in disease pathogenesis [26,27,28].

Zinc also plays a key role in tissue repair and wound healing. As a cofactor for matrix metalloproteinases, it supports keratinocyte migration, extracellular matrix remodeling, and re-epithelialization [29]. In addition, zinc is required for DNA synthesis and cellular proliferation during the proliferative phase of wound healing and promotes keratinocyte differentiation through Human Zinc Transporter Protein 2 (ZIP2), specifically expressed in the epidermis [30]. Consistent with these observations, a randomized double-blind study demonstrated that topical zinc sulfate increases matrix metalloproteinase-1 expression and metallothionein levels within the wound bed [31]. Conversely, zinc deficiency has been associated with impaired immune function and delayed cutaneous repair [15].

## 4. Acne Vulgaris

Acne vulgaris is a chronic inflammatory disease of the pilosebaceous unit, affecting an estimated 9.4% of the global population according to Global Burden of Disease (GBD) data [32,33]. Several tools are currently available for assessing acne severity, among which the Investigator’s Global Assessment (IGA) is one of the most widely used in both clinical trials and routine practice. The IGA is typically based on a 5-point ordinal scale (0–4), ranging from clear skin (grade 0) to severe acne (grade 4), and relies on the investigator’s overall evaluation of inflammatory and non-inflammatory lesions. Another validated instrument is the Global Acne Grading System (GAGS), which assesses acne severity according to both lesion type and anatomical distribution. The resulting composite score enables classification of acne as mild, moderate, severe, or very severe [34]. More broadly, no universally accepted acne severity scoring system currently exists, contributing to heterogeneity in outcome assessment and limiting direct comparisons between clinical trials [35]. Acne vulgaris is a multifactorial inflammatory disorder driven by four interconnected pathogenic processes: increased sebum production, follicular hyperkeratinization, colonization by *C. acnes*, and dysregulated inflammation [36]. Current therapeutic approaches target one or more of these pathways. Topical retinoids primarily normalize follicular keratinization, benzoyl peroxide exerts antimicrobial and comedolytic effects, while topical and systemic antibiotics reduce C. acnes proliferation and inflammation. Hormonal therapies, spironolactone, and isotretinoin act, at least in part, through modulation of sebaceous gland activity and sebum production, with isotretinoin remaining the most effective treatment for severe acne. Pharmacological management therefore relies on a combination of topical and systemic agents selected according to disease severity and clinical presentation, with current strategies emphasizing combination regimens to maximize efficacy, reduce antibiotic exposure, and improve long-term disease control [37].

### 4.1. Biological Rationale for Zinc Use in Acne Vulgaris

Given its broad range of biological activities, zinc has attracted considerable interest as a potential adjunctive or alternative therapeutic option in acne. Experimental and clinical evidence suggests that zinc may influence several key pathogenic pathways simultaneously, including inflammation, innate immune responses, microbial colonization, and sebaceous gland activity. The biological properties of zinc described above may be particularly relevant to inflammatory acne, where its effects on C. acnes-related inflammation, TLR2 signaling, and sebaceous activity provide a plausible therapeutic rationale [12,16,38]. Unlike antibiotics, zinc does not exert the same selective pressure for antimicrobial resistance and may therefore be of interest in antibiotic-sparing strategies [39,40]. Its favorable safety profile, widespread availability, and relatively low cost further support its potential role in long-term disease management. In addition, numerous studies have reported significantly lower serum zinc levels in patients with acne vulgaris compared with healthy controls [41]. Consistent with this biological rationale, a meta-analysis by Yee et al. demonstrated a significant reduction in inflammatory acne lesions following zinc supplementation compared with control groups. However, the benefit appeared limited for non-inflammatory lesions such as comedones, and the overall efficacy of zinc remained inferior to that achieved with standard pharmacological therapies [12].

### 4.2. Clinical Evidence from Early Studies

Early clinical trials conducted in the 1970s produced variable and, at times, conflicting results, largely reflecting differences in study design, zinc formulations, and dosing regimens. In a controlled study, Micheálsson et al. evaluated oral zinc sulfate (providing 135 mg elemental zinc daily), administered alone or in combination with vitamin A, and compared it with vitamin A monotherapy and placebo. Zinc-treated patients showed a significant reduction in inflammatory lesions after four weeks, with no additional benefit observed from the combination with vitamin A. By 12 weeks, acne severity in the zinc monotherapy group had markedly decreased, reaching approximately 13–15% of baseline values. These findings provided early evidence supporting the efficacy of zinc in inflammatory acne, despite the limited understanding of its mechanisms at the time [42]. Other studies conducted during the 1970s reported heterogeneous results, reflecting variability in study design, patient populations, and zinc formulations. In a randomized British trial comparing zinc sulfate with oxytetracycline, both treatments achieved a similar reduction in acne severity of approximately 70% after 12 weeks, with no significant difference in efficacy or adverse effects between groups [43]. However, other studies did not demonstrate a clear advantage of zinc over placebo for certain lesion types, particularly non-inflammatory lesions, likely due to differences in dosing and study populations [44,45]. Overall, by the end of the 1970s, zinc was generally recognized as having therapeutic activity in inflammatory acne, although the magnitude of benefit appeared inconsistent across studies.

### 4.3. Formulation and Dose

In studies investigating the role of zinc in acne, common oral doses have ranged from 30 mg to 135 mg of elemental zinc per day. Early trials mainly used high doses of zinc sulfate (approximately 600 mg daily, corresponding to about 135 mg elemental zinc), which were frequently associated with gastrointestinal adverse effects [45,46]. Gastrointestinal intolerance emerged as a major limitation, with some studies reporting adverse events in up to 40% of patients and treatment discontinuation in around 20% because of severe nausea. These findings highlighted the dose-dependent toxicity of zinc sulfate and prompted investigation into alternative zinc salts, including zinc gluconate and zinc acetate, which were hypothesized to achieve therapeutic effects at lower elemental doses with improved tolerability [47]. Placebo-controlled clinical trials conducted during the 1980s and 1990s evaluated zinc gluconate and zinc sulfate separately in patients with acne. While zinc sulfate has been associated with a relatively high frequency of gastrointestinal adverse effects and zinc gluconate has generally shown a favorable tolerability profile, direct comparative studies between the two zinc salts are lacking. As a result, any suggestion that zinc gluconate is better tolerated than zinc sulfate should be interpreted with caution, as it is supported mainly by indirect evidence rather than by head-to-head clinical trials [47,48]. Overall, the available evidence indicates that the clinical utility of zinc depends not only on efficacy but also on achieving an optimal balance between therapeutic benefit, bioavailability, and tolerability. Moderate dosing regimens appear to provide meaningful clinical improvement while minimizing the risk of dose-related adverse effects, particularly gastrointestinal symptoms, and are therefore generally preferred in clinical practice. Similarly, although zinc absorption is greatest when administered on an empty stomach, intake with meals is often recommended to improve treatment adherence and gastrointestinal tolerability. The bioavailability of zinc may nevertheless be influenced by dietary factors, with phytate-rich foods known to reduce intestinal absorption [49].

Although short-term zinc therapy is generally well tolerated, prolonged high-dose supplementation requires caution because zinc and copper compete for intestinal absorption. At intakes of approximately 100–300 mg elemental zinc daily, chronic supplementation has been associated with copper deficiency, potentially leading to anemia, neutropenia, and impaired immune function [50,51]. Reassuringly, most acne treatment regimens involve relatively short courses of therapy, typically lasting 6–13 weeks, which are generally considered low-risk [38]. For patients requiring prolonged supplementation, some authors recommend periodic monitoring or concurrent copper supplementation [52]. Taken together, the available data support a favorable benefit-risk profile for oral zinc in acne management [12].

### 4.4. Comparative Studies and Meta-Analyses

As better-tolerated zinc formulations became available and clinical experience accumulated, interest in zinc as a therapeutic option for acne was renewed. Several controlled studies subsequently evaluated oral zinc both as monotherapy and in combination with established treatments, frequently comparing its efficacy with that of systemic antibiotics. The main characteristics and outcomes of these studies are summarized in Table 1. Interest in oral zinc therapy was further reinforced by a large multicenter randomized double-blind trial conducted by Dréno et al. in 2001 [53], which compared 30 mg of elemental zinc administered as zinc gluconate daily with 100 mg minocycline daily in 332 patients with inflammatory acne vulgaris. After three months of treatment, both groups showed clinical improvement; however, minocycline achieved significantly greater reductions in inflammatory lesions and higher rates of clinical success, defined as at least a two-thirds reduction in inflammatory lesion count. Clinical success was observed in 63.4% of patients treated with minocycline compared with 31.2% of those receiving zinc gluconate. Nevertheless, zinc treatment still produced meaningful improvement in approximately one-third of patients and was associated with a tolerability profile comparable to that of minocycline, with mainly mild gastrointestinal adverse effects reported in both groups. These findings suggested that oral zinc gluconate may represent a reasonable alternative in selected patients with mild-to-moderate inflammatory acne, particularly when systemic antibiotics are poorly tolerated or contraindicated [53]. In 2013, Brandt conducted a systematic review of the literature on zinc for acne treatment, evaluating both oral and topical formulations according to Strength of Recommendation Taxonomy criteria, commonly known as SORT. The SORT system is a clinical grading taxonomy used to evaluate the quality of medical evidence, where recommendations are rated from A for consistent and high-quality evidence to C for consensus or disease-oriented evidence [54]. Based on this framework, the review assigned a SORT strength of recommendation of B, which indicates inconsistent or limited-quality patient-oriented evidence, to both oral and topical zinc. This evaluation noted that the preponderance of evidence suggests zinc has antibacterial and anti-inflammatory effects and may reduce sebum production [55]. Similarly, a later review by Cervantes et al. analyzed 12 clinical studies evaluating zinc in acne vulgaris, most of which were randomized placebo-controlled trials investigating oral zinc supplementation. Eight studies demonstrated significant improvements in acne severity, particularly regarding inflammatory lesions, whereas four failed to show significant benefit over placebo. The authors emphasized that discrepancies among studies were likely related to small sample sizes, heterogeneous patient populations, and differences in zinc formulations and dosages [56].

More recently, Yee et al. performed a comprehensive systematic review and meta-analysis including 25 studies and 2445 participants. Observational studies included in the analysis demonstrated significantly lower serum zinc levels in patients with acne vulgaris compared with healthy controls, supporting the hypothesis that altered zinc homeostasis may contribute to acne pathogenesis in at least a subset of patients. Interventional studies further showed that zinc supplementation significantly reduced inflammatory papule counts, while no consistent benefit was observed for non-inflammatory lesions such as comedones. Importantly, no significant increase in adverse effects was detected in zinc-treated patients compared with control groups, suggesting that zinc supplementation at commonly used doses is generally safe and well tolerated [12].

### 4.5. Comparative Efficacy of Oral Zinc and Conventional Therapies in Acne Vulgaris

Growing concerns about antibiotic resistance have renewed interest in zinc as a potential alternative to systemic antibiotics for acne treatment. In an open-label randomized study, Tolino et al. compared oral zinc sulfate (400 mg daily) with lymecycline in 100 patients with mild-to-moderate papulopustular acne over a 12-week period. Acne severity was assessed using the GAGS. Both treatment groups showed significant reductions in acne severity scores after 4 and 12 weeks of therapy, suggesting that zinc sulfate may achieve clinical improvements comparable to antibiotic therapy. Patients receiving zinc also reported greater improvements in acne-related quality of life, as measured by the Acne Quality of Life questionnaire, a validated acne-specific 19-item instrument evaluating self-perception, social functioning, emotional well-being, and acne symptoms. Nevertheless, these findings should be interpreted cautiously. The study employed an open-label design and relied on the partly subjective GAGS scoring system rather than objective lesion counts, factors that may have introduced assessment bias. Furthermore, the relatively small sample size and the absence of a placebo arm limit the robustness of the conclusions. Although the findings support zinc sulfate as a promising antibiotic-sparing option for mild-to-moderate inflammatory acne, larger double-blind randomized controlled trials are still needed before it can be considered truly equivalent to systemic tetracycline-class antibiotics in routine clinical practice [57]. More recently, interest has shifted toward the use of zinc as an adjunct to isotretinoin therapy. In 2022, Salah et al. conducted a preliminary trial where one group of acne patients received standard-dose isotretinoin (0.5 mg/kg/d) while another group received low-dose isotretinoin (0.25 mg/kg/d) plus oral zinc. The results showed no difference in acne clearance between the two groups, but the combination group experienced markedly fewer side effects, with only 20% having typical isotretinoin-related adverse effects, such as cheilitis, xerosis, epistaxis, dry eyes, alopecia, and musculoskeletal discomfort, versus 76.7% in the high-dose isotretinoin group. Relapse rates after treatment were similar for both regimens. These findings suggest that zinc supplementation may enhance the tolerability of isotretinoin treatment, potentially allowing lower isotretinoin doses to be used without compromising efficacy. Although the available evidence remains preliminary, zinc may represent a promising isotretinoin-sparing strategy for patients who experience dose-limiting adverse effects or are unable to tolerate conventional dosing regimens. Further studies are required to confirm these observations and to define the optimal role of zinc in combination therapy [58].

### 4.6. Current Consensus

Despite the growing body of evidence, oral zinc is not currently considered a first-line treatment in most acne management guidelines. This is due to the inconsistent findings and methodological limitations that characterized many of the earlier studies, including small sample sizes and substantial heterogeneity in study design [38]. Nevertheless, the role of zinc in acne treatment has become increasingly clear over the past decade. Recent dermatology reviews recognize oral zinc, particularly zinc sulfate and zinc gluconate, as a useful option for inflammatory acne, with the most consistent benefits observed in the reduction of papules and pustules rather than comedonal lesions [16]. Meta-analyses have also demonstrated that acne patients tend to have significantly lower serum zinc levels compared with healthy controls, suggesting that altered zinc homeostasis may contribute to acne pathogenesis in at least a subset of patients and supporting the potential value of assessing zinc status in recalcitrant or inflammatory acne [12]. While it is unlikely to replace retinoids or isotretinoin in severe nodulocystic acne, it may serve as an adjunctive or alternative treatment in mild-to-moderate inflammatory acne, especially for patients who cannot tolerate systemic antibiotics or for whom reducing long-term antibiotic exposure is a therapeutic priority [39]. Recent clinical studies exploring zinc as an antibiotic-sparing or isotretinoin-sparing strategy further support this evolving role. Importantly, available evidence suggests that oral zinc is generally well tolerated, with most studies reporting only mild gastrointestinal adverse effects and no significant increase in side effects compared with control or comparator treatments [12,38]. This favorable safety profile makes zinc particularly attractive for adolescents and young adults seeking less aggressive systemic therapies. Furthermore, a randomized controlled trial involving subjects aged 13 to 40 years reported no adverse events with a combination containing lactoferrin, vitamin E, and zinc, further supporting the tolerability of zinc-containing regimens [59].

Taken together, the available evidence supports a potential role for zinc in the management of acne; however, substantial heterogeneity in study design, zinc formulation and dosage, treatment duration, comparator groups, and outcome measures limits direct comparisons across studies and prevents definitive conclusions regarding its efficacy and optimal clinical use.

## 5. Hidradenitis Suppurativa

HS is a chronic, relapsing inflammatory disease of the hair follicle that manifests as painful nodules, abscesses, and draining sinus tracts, typically in intertriginous areas rich in apocrine glands, such as axillae, groin, perineum, and inframammary folds. The disease affects approximately 0.40% of the general population [60]. The pathogenesis of HS is complex and multifactorial. It is generally believed to begin with follicular hyperkeratinization, leading to follicular occlusion and subsequent rupture of the follicular unit. The release of keratin, cellular debris, and microbial products into the dermis then triggers an exaggerated inflammatory response [2,61,62]. HS skin exhibits abnormalities in innate immunity, including reduced expression of antimicrobial peptides such as defensins and impaired epidermal barrier function, which may facilitate microbial persistence and contribute to alterations in the cutaneous microbiome [63,64]. HS lesions frequently harbor increased levels of anaerobic bacteria such as Prevotella, Porphyromonas, Fusobacterium, and Proteus species, as well as Staphylococcus spp. Although their precise pathogenic role remains incompletely understood, these microbial communities may contribute to the persistence of inflammation [65,66,67]. Dysregulation of both innate and adaptive immunity, with increased expression of pro-inflammatory cytokines including TNF-α, interleukin IL-1β, IL-17, and IL-23, plays a central role in sustaining chronic inflammation and promoting fistula formation [2,68,69]. Clinically, disease severity is most commonly assessed using the Hurley staging system, which classifies HS into three stages according to the presence and extent of recurrent abscesses, sinus tract formation, and scarring [70], while the International Hidradenitis Suppurativa Severity Score System (IHS4) provides a validated dynamic tool for assessing inflammatory burden and monitoring treatment response [71]. More recently, the IHS4-55, defined as a ≥55% reduction in the IHS4 score, has been proposed and externally validated as a clinically meaningful outcome measure for evaluating therapeutic response in both clinical trials and real-world settings [72]. Current management of HS requires a multimodal approach tailored to disease severity and includes lifestyle modification, antibiotic therapy, biologic treatment targeting TNF-α and IL-17, and surgical interventions [2,73]. In this scenario, high-resolution skin ultrasound combined with color-Doppler provides objective monitoring of the reduction in vascularization and inflammation during chronic medical treatments, overcoming the sensitivity limitations of purely clinical assessments [74]. The syndromic forms of the disease, such as PASH or PAPASH syndromes, demonstrate how can HS be linked to precise genetic profiles often accompanied by systemic comorbidities, including joint and gut inflammation [75].

In addition to immune and microbial mechanisms, hormonal factors, particularly androgens, have also been implicated in HS pathogenesis. Clinical observations suggest a hormonal contribution, as the disease typically appears after puberty, often fluctuates during pregnancy, and may improve after menopause. Although hormonal factors appear to influence disease activity, serum androgen levels are not elevated in HS patients, supporting the hypothesis of enhanced end-organ sensitivity rather than systemic hormonal abnormalities [76,77]. Among modifiable risk factors, cigarette smoking and obesity show the strongest associations with HS. Up to 80% of patients are current or former smokers, and smoking is consistently associated with a higher disease incidence compared with non-smokers. Obesity is likewise strongly linked to both disease prevalence and severity, with increasing body mass index correlating with a greater clinical burden. Beyond observational evidence, Mendelian randomization studies support a causal relationship between elevated body mass index and HS, whereas the causal role of smoking remains less clearly established [78]. HS is also frequently associated with other immune-mediated inflammatory diseases, including pyoderma gangrenosum, ankylosing spondylitis, Crohn’s disease, and ulcerative colitis, highlighting the systemic inflammatory nature of the disease [79].

### 5.1. Biological Rationale for Zinc Use in Hidradenitis Suppurativa

A multicenter case–control study by Poveda et al. in 2018 demonstrated that low serum zinc levels are significantly more prevalent in HS patients compared to healthy controls [13]. Moreover, reduced zinc levels were associated with greater disease severity, including Hurley stage III disease, involvement of three or more anatomical sites, and genital or perineal localization. These findings suggest that zinc deficiency may contribute to the impaired innate immune response and heightened inflammatory activity characteristic of HS [13].

The observation of reduced zinc levels in HS patients has stimulated interest in zinc as a potential therapeutic approach. These findings have stimulated interest in zinc as a potential therapeutic approach in HS, particularly because of its effects on innate immune regulation [21,80]. In a prospective experimental study, Dréno et al. demonstrated that oral zinc administration for three months upregulated the expression of antimicrobial peptides and innate immunity-related genes in lesional skin, suggesting that zinc supplementation may partially restore the innate immune dysfunction observed in HS [22].

Zinc may also affect microbial factors implicated in disease persistence [25]. Although HS is not a primary infectious disease, bacterial colonization and biofilm formation within sinus tracts are thought to exacerbate chronic inflammation and disease persistence [81,82]. Biofilms provide a protective environment that promotes bacterial survival and resistance to host immune defenses. Interestingly, zinc has been shown in vitro to inhibit bacterial biofilm formation, raising the possibility that part of its therapeutic benefit in HS may be derived from modulation of the cutaneous microbiome and reduction in biofilm-associated inflammation [25].

Hormonal influences may further support the rationale for zinc therapy in HS. The disease typically develops after puberty and frequently fluctuates during pregnancy, the menstrual cycle, or menopause, suggesting a role for androgen-mediated mechanisms [83]. Clinical improvement observed in observational studies and case series involving anti-androgen therapies such as spironolactone and finasteride further supports this hypothesis. Zinc has been reported in experimental biochemical studies to modulate 5α-reductase activity, potentially decreasing the conversion of testosterone to dihydrotestosterone in the skin and consequently limiting follicular occlusion and downstream inflammation [26].

Another potentially beneficial aspect of zinc therapy relates to tissue repair. Chronic HS lesions frequently heal poorly and may result in persistent ulcers, sinus tracts, and scarring. Zinc plays an essential role in collagen synthesis, keratinocyte migration, and extracellular matrix remodeling, acting as a cofactor for several enzymes involved in wound healing, including matrix metalloproteinases [29,84].

Taken together, these observations indicate that zinc could influence multiple pathways involved in HS pathogenesis, including immune dysregulation, microbial persistence, hormonal signaling, and tissue repair. However, much of the available evidence remains mechanistic or experimental, and further well-designed clinical studies are needed to establish the extent to which these biological effects translate into meaningful therapeutic outcomes for patients with HS.

### 5.2. Clinical Evidence from Studies in Hidradenitis Suppurativa

The therapeutic potential of oral zinc in HS was first investigated in the mid-2000s and has since been explored in several observational studies and small clinical trials, the main characteristics of which are summarized in Table 2.

In a pilot prospective open-label study published in 2007, Brocard et al. investigated oral zinc gluconate 90 mg daily for 4 months in 22 patients with mild-to-moderate HS refractory to conventional treatments. All participants experienced some degree of clinical improvement, with complete remission achieved in eight patients and substantial partial responses observed in the remainder. Following remission, most patients were able to reduce the zinc dosage while maintaining disease control. Treatment was generally well tolerated, with only mild gastrointestinal adverse events reported, including diarrhea, abdominal pain, and esophagitis. Only one patient discontinued therapy during the study period. These preliminary results suggested that zinc salts could represent a useful therapeutic option in a disease often characterized by chronicity and resistance to treatment [85].

Further support for the anti-inflammatory effects of zinc was provided by Hessam et al., who retrospectively evaluated zinc gluconate 90 mg/day in combination with topical triclosan 2% in 66 patients with Hurley stage I–II HS. Over 3 months, patients showed significant reductions in inflammatory nodules and new flares. However, fistula count showed no significant difference, confirming that zinc + triclosan helps control active inflammation but cannot reverse pre-existing sinus tracts. Side effects, mostly abdominal pain and nausea, were reported by 12 patients. The authors concluded that this combination can be considered an anti-inflammatory treatment for initial HS [86].

The potential role of zinc has also been explored in pediatric HS. Offidani et al. reported outcomes in eight prepubertal patients treated with a combination of oral zinc supplementation, azithromycin, and topical clindamycin. During the 52-week follow-up, patients receiving the combined regimen experienced significant improvements in clinical severity, pain, and quality of life, whereas zinc supplementation without azithromycin showed less pronounced benefits. These findings suggest that zinc may represent a useful adjunctive therapy in pediatric HS, particularly when long-term treatment options are limited. The authors also stressed the need for dedicated pediatric guidelines, as HS in this age group is uncommon and often associated with hormonal disorders and genetic predisposition [87].

More recently, Molinelli et al. performed a retrospective maintenance therapy study evaluating zinc gluconate 90 mg/day combined with nicotinamide after oral minocycline treatment in patients with Hurley stage I–II HS. Compared with untreated controls, over 6 months of follow-up, the zinc/nicotinamide group had significantly fewer HS flares and longer flare-free intervals. At both 12 and 24 weeks after antibiotics, the treated group showed a reduced number of new nodules/abscesses, shorter flare duration, and improved quality-of-life scores, such as lower pain Visual Analog Scale and Dermatology Life Quality Index, compared to controls. By 24 weeks, the control group averaged approximately 9 acute flares versus 4 in the zinc group. Disease severity scores evaluated by IHS4 remained nearly stable in the zinc group but worsened in the controls. Tolerability was excellent, with only two patients reporting mild nausea. The authors concluded that zinc with nicotinamide represents a valuable, well-tolerated maintenance approach for mild–moderate HS.

In HS, the evidence is considerably more limited than in acne and is mainly based on small observational or uncontrolled studies. Although clinical improvement has been reported, these preliminary findings suggest that zinc may have a potential role as a maintenance strategy following initial disease control, possibly contributing to a reduction in flare frequency and mitigating the recurrent nature of HS [88].

However, the lack of adequately powered randomized controlled trials prevents firm conclusions regarding its efficacy and its optimal place in the therapeutic algorithm.

### 5.3. Dosing in Hidradenitis Suppurativa

Current evidence suggests that oral zinc gluconate at a dose of 90 mg/day, the regimen most commonly evaluated in available studies, may reduce disease severity and inflammatory activity in patients with mild-to-moderate HS [85,86]. However, despite these promising results, evidence remains limited, and further controlled studies are required to establish the optimal formulation, dosage, and duration of zinc therapy in HS.

## 6. Safety

Across studies investigating zinc supplementation in both acne vulgaris and HS, treatment has generally been reported as safe and well tolerated, with adverse effects being predominantly mild and gastrointestinal in nature [89]. The most frequently reported side effects include nausea, abdominal discomfort, gastric irritation, and dyspepsia occurring in approximately 20% of patients [85,86].

An important safety concern related to prolonged high-dose zinc supplementation is the risk of zinc-induced copper deficiency (ZICD) caused by competitive inhibition of intestinal copper absorption. Early manifestations of copper deficiency are typically hematologic and include anemia and neutropenia, whereas prolonged deficiency may result in severe neurological complications such as sensory ataxia, peripheral neuropathy, gait disturbances, and myelopathy, which may be only partially reversible despite treatment [50,90]. Recent studies suggest that ZICD is likely underrecognized in clinical practice. In a study by Duncan et al., approximately half of the patients with confirmed ZICD had initially remained undiagnosed, with the diagnosis being established only after repeat laboratory testing identified concomitant hyperzincemia and hypocupremia [91]. In a retrospective analysis, approximately 13% of patients receiving high-dose zinc supplementation developed hematological and/or neurological manifestations compatible with copper deficiency, although biochemical confirmation was often lacking [51]. Because zinc is frequently administered for prolonged periods in inflammatory dermatologic diseases such as acne vulgaris and HS, periodic monitoring of complete blood count and serum copper levels should be considered during long-term or high-dose therapy. Although no standardized monitoring schedule has been established specifically for acne or HS, baseline assessment followed by periodic monitoring. The authors suggest to dose copper levels every 2–3 months during prolonged high-dose supplementation. Earlier laboratory assessment should be performed in patients developing otherwise unexplained anemia, neutropenia, or neurological symptoms suggestive of copper deficiency [92].

## 7. Place in Therapy and Future Directions

Although zinc shares some anti-inflammatory effects in acne and HS, the mechanisms underlying its clinical benefit may not be the same in the two conditions. In acne, its activity appears to be mainly related to the pilosebaceous unit, including the modulation of sebaceous activity and 5α-reductase, as well as its effects on *C. acnes* and TLR2-mediated inflammation [21,26]. In HS, where sebaceous activity and *C. acnes* have a less prominent role, zinc may be more relevant for its effects on innate immunity, antimicrobial peptide expression, and microbial persistence and biofilm formation [22,25]. These differences may explain why the relative contribution of the anti-inflammatory and antimicrobial properties of zinc varies between acne and HS.

Zinc may also exert part of its biological effects through modulation of host–microbiome interactions. Zinc availability can influence microbial growth and competition, thereby affecting gut microbial composition. In addition, adequate zinc status contributes to intestinal barrier integrity and mucosal immune homeostasis, potentially limiting the translocation of bacteria and microbial products and the resulting inflammatory response [93]. Dysbiosis of the skin and gut microbiome has been implicated in the pathogenesis of both acne and HS [94,95], suggesting a possible link between zinc status, host–microbiome interactions, and cutaneous inflammation. However, the effects of zinc on microbial communities appear to be complex and dose-dependent, and direct evidence that oral zinc improves acne or HS through microbiome modulation is currently lacking.

Despite the growing interest in zinc as a therapeutic option for HS, the current evidence base remains limited. Most available data derive from small open-label studies, retrospective cohorts, and only a few controlled clinical investigations. As a result, the precise role of zinc within HS management has yet to be clearly established, and larger randomized controlled trials are needed to determine its efficacy, optimal dosage, and duration of treatment [96]. At present, it also remains unclear whether zinc supplementation may alter disease progression or provide long-term preventive benefits, particularly in early-stage HS. Expectations should therefore be carefully managed, especially in patients with longstanding and severe disease (Hurley stage III), in whom extensive fibrosis and scarring often require biologic therapy or surgical intervention [97]. Nevertheless, zinc may still represent a useful adjunctive treatment for controlling inflammation and reducing flare frequency.

Interpretation of the available evidence is limited by the considerable heterogeneity across studies. Differences involve zinc formulation and dosage, treatment duration, disease severity, concomitant therapies, comparators, and clinical outcome measures. Moreover, the distinction between zinc salt dose and elemental zinc dose is not always clearly reported, further limiting comparisons between studies. These methodological differences make it difficult to define the optimal formulation and dose of zinc, as well as the patient population most likely to benefit from supplementation. Differences among oral zinc formulations may further contribute to this heterogeneity, as zinc salts differ in elemental zinc content and may also differ in absorption and bioavailability. Comparative human data, however, remain limited [98]. The main characteristics of commonly used oral zinc formulations are summarized in Table 3.

Another unresolved issue is the potential influence of baseline zinc status on treatment response. Although lower serum zinc levels have been reported in patients with acne and HS [12], it remains unclear whether supplementation is more effective in zinc-deficient patients or may also provide benefits in patients with adequate zinc status. Moreover, serum zinc concentrations should be interpreted with caution, as inflammation may reduce circulating zinc levels independently of total body zinc stores [99]. Future studies should therefore assess baseline zinc status and investigate whether it may help identify patients more likely to benefit from supplementation.

Current clinical guidelines reflect the different levels of evidence supporting oral zinc in acne and HS. In acne, oral zinc is not included among the treatments recommended by the recent American Academy of Dermatology guidelines, despite the potential benefits reported in previous clinical studies [100]. In contrast, the European S2k guidelines for HS include zinc gluconate as a possible second-line maintenance treatment for mild-to-moderate disease and also consider supplementation in zinc-deficient patients [7]. Overall, these recommendations support a selective and adjunctive role for oral zinc rather than its use as a replacement for established first-line therapies.

## 8. Conclusions

Oral zinc supplementation has emerged as a potential adjunctive therapeutic option for both acne vulgaris and HS, supported by its well-established antibacterial, anti-inflammatory, immunomodulatory, and antioxidant properties. Based on the available evidence, oral zinc may be considered as an adjunctive option in selected patients with mild-to-moderate acne, particularly when conventional therapies are contraindicated or poorly tolerated, or when prolonged antibiotic use is undesirable. In HS, preliminary clinical evidence suggests a potential benefit of zinc supplementation, particularly in mild-to-moderate disease. Zinc gluconate has been associated with reductions in disease severity, flare frequency, and inflammatory activity, with potential benefit reported particularly in patients with Hurley stage I–II disease and when used as maintenance or adjunctive therapy [80,88]. However, the available evidence is derived predominantly from small observational or uncontrolled studies, with a lack of adequately powered randomized controlled trials. Therefore, zinc should currently be regarded as an adjunctive option rather than an established stand-alone treatment, particularly in moderate-to-severe HS, where systemic and biologic treatments remain essential [80,101]. Given its generally favorable safety profile, low cost, and accessibility, zinc may nevertheless represent a potential adjunctive strategy in selected patients.

## Figures and Tables

**Table 1:** Main Clinical Evidence on Oral Zinc Supplementation in Acne Vulgaris. Key clinical evidence supporting the use of oral zinc in acne vulgaris. The included studies comprise randomized controlled trials, systematic reviews, and meta-analyses.

Study	Year	Study Design	Population	Zinc Formulation/Dose	Elemental Zinc Dose	Treatment Duration	Comparator	Main Findings	Main Limitations
Dréno et al. [53].	2001	Multicenter randomized double-blind controlled trial	332 patients with inflammatory acne vulgaris	Zinc gluconate	30 mg/day	3 months	Minocycline 100 mg/day	Both groups improved; minocycline showed greater efficacy, but zinc achieved improvement in approximately one-third of patients with similar tolerability	Absence of placebo control
Cervantes et al. [56].	2018	Literature review	12 clinical studies on zinc supplementation in acne	Multiple oral zinc formulations and regimens	Variable across included studies	Variable across included studies	Placebo or standard therapies	Eight studies demonstrated significant improvement, mainly in inflammatory lesions	Small sample sizes and methodological variability
Yee et al. [12].	2020	Systematic review and meta-analysis	25 studies; 2445 participants	Multiple zinc formulations and doses	Variable across included studies	Variable across included studies	Control groups/placebo	Significant reduction in inflammatory papules; acne patients had lower serum zinc levels compared with controls	High heterogeneity and inconsistent effects on non-inflammatory lesions
Tolino et al. [57].	2021	Controlled retrospective clinical study	100 patients affected by mild to moderate acne vulgaris	Zinc sulfate 400 mg/day	Not clearly reported	12 weeks	300 mg tablet one daily of lymecycline.	Zinc sulphate showed efficacy and tolerability comparable to lymecycline	Open-label study design and reliance on subjective acne severity assessment

**Table 2:** **Main Clinical Studies Evaluating Zinc Supplementation in Hidradenitis Suppurativa**. *Main clinical studies evaluating oral zinc supplementation in hidradenitis suppurativa*. *The available evidence consists primarily of observational studies, pilot clinical investigations*, *and retrospective analyses*.

Study	Year	Study Design	Population	Zinc Formulation/Dose	Treatment Duration	Comparator	Main Findings	Main Limitations
Brocard et al. [85].	2007	Prospective open-label pilot study	22 patients with mild-to-moderate HS refractory to conventional treatments	Zinc gluconate 90 mg/day	4 months	None	Complete remission was achieved in 8 patients, while the remaining patients showed marked improvement.	Small sample size, absence of a control group, open-label design, and limited external validity.
Hessam et al. [86].	2016	Retrospective observational study	66 patients with Hurley stage I–II HS	Zinc gluconate 90 mg/day	3 months	Baseline disease status	Significant reductions were observed in inflammatory nodules and new flares.	Retrospective design, lack of placebo; concomitant topical triclosan.
Offidani et al. [87].	2019	Observational case series	8 prepubertal children with HS	Oral zinc supplementation combined with azithromycin and topical clindamycin	Maintenance treatment up to 52 weeks	None	These findings suggest that zinc may serve as a useful adjunctive therapy in pediatric HS.	Very small sample size, pediatric-only population, observational design; concomitant treatments.
Molinelli et al. [88]	2020	Retrospective maintenance therapy study	92 patients with Hurley stage I–II HS previously treated with oral minocycline	Zinc gluconate 90 mg/day + nicotinamide 30 mg/day	90 days	Untreated controls	Patients receiving zinc and nicotinamide showed fewer and shorter flares, fewer new inflammatory lesions, longer remission periods, and improved quality of life.	Retrospective design, non-randomized, concomitant nicotinamide treatment.

**Table 3:** Comparison of commonly used oral zinc formulations, including approximate elemental zinc content, doses reported in acne vulgaris and hidradenitis suppurativa studies, bioavailability, and gastrointestinal tolerability.

Zinc Formulation	Approximate Elemental Zinc Content *	Doses Reported in Acne/HS Studies	Bioavailability	Gastrointestinal Tolerability
**Zinc gluconate**	~14% [52,97]	Acne: zinc gluconate 200 mg/day [53]; HS: zinc gluconate 90 mg/day [85,86,88]	Water-soluble; human studies suggest favorable absorption, although direct comparative evidence remains limited [97]	Generally well tolerated [86,97]
**Zinc sulfate**	~23% [52,97]	Acne: 400 mg/day [57]	Water-soluble and orally absorbed; direct comparative human evidence between zinc formulations remains limited [97]	Gastrointestinal adverse effects may occur [57,97]
**Zinc acetate**	~30% [52,97]	Not used in evalueted studies	Water-soluble; direct comparative evidence with other zinc salts in humans remains limited [97]	Gastrointestinal adverse effects may occur [97]

* Note: Doses are reported according to the zinc formulation used in the original studies rather than as calculated elemental zinc doses. Elemental zinc percentages are approximate and may vary according to the specific chemical form and formulation.

## Data Availability

No new data were created or analyzed in this study. Data sharing is not applicable to this article.
